# Non-technical Skills for Medical Students: Validating the Tools of the Trade

**DOI:** 10.7759/cureus.24776

**Published:** 2022-05-06

**Authors:** Lysander J Gourbault, Erin L Hopley, Francesca Finch, Sally Shiels, Helen Higham

**Affiliations:** 1 Oxford Simulation, Training and Research (OxSTaR), John Radcliffe Hospital, Oxford, GBR

**Keywords:** human factors, validation study, skills and simulation training, medical school education, non-technical skills

## Abstract

The Medical Students’ Non-Technical Skills (Medi-StuNTS) is a behavioural marker scheme (BMS) designed to assess non-technical skills (NTS) in medical students in emergency simulations. This study aimed to assess the evidence for validity and usability of Medi-StuNTS by naive, near-peer educators. Nine doctors assessed four students in simulations of common medical emergencies. The scores were used to assess inter-rater reliability, inter-class correlation, and observability. Students and assessors completed questionnaires that assessed the tool’s usability and consequence.

Inter-rater agreement across all skill elements was “high” with rWG scores >0.8. An inter-class correlation was “good” with ICC3K kappa scores of 0.86 and 0.89 overall, when measured per simulation and per skills element respectively. Overall skill observability was high (>80%) except for coping with stress. Assessors found the tool “difficult to use” but “useful for feeding back in a constructive way”. Students appreciated the comprehensiveness of the feedback as well as knowing what to expect during debriefs.

This study has shown that the Medi-StuNTS BMS has good usability and evidence of validity in naive assessors and near-peer educators. It shows the particularly good internal structure and overall beneficial consequences. Further study will be necessary to understand how best to deploy it in formative and summative contexts.

## Introduction

In order to deliver safe and effective care to patients, a combination of both technical and non-technical skills is required [1]. Non-technical skills (NTS) are defined as the cognitive, social and personal resource skills that complement technical skills, and contribute to safe and efficient task performance [2]. Errors in NTS are known to increase the risk of adverse events occurring in the workplace [2]. It is therefore important to train current and future clinicians in these skills in order to deliver safe and effective patient care. The teaching of NTS has been broadly lacking in UK undergraduate medical education - it was only in 2018 that it became a requirement for medical schools to teach on the subject as outlined in the GMC’s new “Outcomes for Graduates” [3].

In order to successfully establish NTS into undergraduate training, their assessment needs to be considered. Assessment is a tool not just of learning but for learning - it allows the student and teacher to optimise their capabilities [4]. This is no different from the assessment of NTS, where Behavioural Marker Schemes (BMS) are used. BMS are based on the observable NTS that contributes to performance in a specific context [5]. The most commonly included skill domains are communication, teamwork, leadership, and decision-making [6].

Like all assessment tools, it is essential that each is tailored to the appropriate specialty and stage of training [1]. Over the years, over 70 different NTS assessment tools have been created in healthcare. This has led to challenges for educators in choosing the most appropriate one [6]. Specialties like anaesthetics and surgery have established tools such as Anaesthetists’ Non-Technical Skills (ANTS) and Non-Technical Skills for Surgeons (NOTSS), which have moved to the forefront after being extensively validated [1, 7-9]. To our knowledge, only three BMS targeted specifically at medical student training exist with none having yet taken the ascendancy in this way [6]. In order to avoid further multiplicity of tools, we looked to assess the evidence of the validity of the existing Medical Students’ Non-Technical Skills (Medi-StuNTS). Unlike other BMS available, Medi-StuNTS is tailored to train and assess NTS in UK medical students, focussing particularly on emergency simulations.

Moreover, with the introduction of simulation in medical student examinations, it is essential that the assessment tools used have good evidence of validity. The modern approach to validity is to construct a “validity argument” that provides evidence from five sources: content, response process, internal structure, relations with other variables and consequences [10,11]. The original paper by Hamilton et al. describes the systematic creation of the Medi-StuNTS tool by subject matter experts - this provides evidence of its content validity [1]. They have further assessed their tool’s internal structure and relation to other variables in their recent paper, which uses two experienced assessors [12].

However, to date, no evidence exists on the tool’s performance when used by naive assessors or by near-peer educators. This is important as near-peer teaching forms an increasing, and often unaccredited, part of medical education [13] and, although effective educators, near-peer colleagues have little experience in using BMS [14].

This study aimed to further assess the construct validity and usability of Medi-StuNTS by near-peer educators for assessing NTS in final year medical student simulations. We focused on internal structure - measured by inter-rater reliability and inter class correlation. Additionally, we assessed the tools usability for simulation assessment as well as a framework to structure simulation debriefs.

## Materials and methods

Ethical approval

Ethical approval was obtained from Oxford University Hospitals Clinical Trials and Research Governance.

Simulation session

Assessors

Nine doctors working at the John Radcliffe Hospital, Oxford, with more than two years of clinical experience post qualification were recruited to be assessors. These were recruited via an advertisement email to all doctors in training at the John Radcliffe Hospital who had the required clinical experience. They were asked to complete the pre-reading material provided by the Medi-StuNTS creators on the tool’s background and how to use it. This was expected to take one and a half hours to complete. Before the simulation session assessors received a briefing regarding the tool and any questions were answered. This took a further half an hour, bringing the total training time to two hours.

Running of Simulation Sessions

Four Oxford University final year medical students were recruited to take part in four simulation scenarios. These were recruited via an advertisement email to all final-year medical students studying at Oxford University. Their participation was entirely voluntary with no financial incentive. To ensure the students were being assessed on skills they had been taught, they all took part in a two-hour NTS teaching course.

The medical students worked in pairs with one taking the lead (the leader), and the other following (the follower). Simulation scenarios were selected from those run by Oxford University for final year students during their Emergency Medicine rotations. These cover some of the most common medical emergencies seen by newly qualified doctors [1,3,15]. The simulations were adapted to incorporate situations that tested key NTS. These included the use of faculty as a confederate presenting challenging behaviour, the requirement to escalate concerns, unclear diagnosis and faulty equipment.

Simulation sessions took place in a classroom using a low fidelity results manikin. The students were familiarised with the equipment available to them prior to starting the simulation.

Each scenario lasted 10 minutes with 15 minutes given to debrief students. One member of the faculty acted as a nurse helping the students, one ran the simulation, and another led the student to debrief.

At the end of each simulation, the Medi-StuNTS BMS was used to debrief students providing a framework to discuss key NTS. The students were not given their specific scores as recommended by the Medi-StuNTS authors.

Data Collection

Before and after the simulations the students were given a questionnaire to complete. Using this they rated their understanding of NTS and confidence in dealing with emergency scenarios. They also provided feedback on the session (Appendix A).

Likewise, the assessors were asked to provide feedback on the usability and acceptability of the Medi-StuNTS tool (Appendix B).

Assessors watched each simulation scenario, and using the Medi-StuNTS tool, they scored both the leader and follower on their NTS. Assessment scores were then collected anonymously and analysed.

Data analysis

Questionnaire data were assessed using basic descriptive statistics and we conducted a thematic analysis of the qualitative data presented in tables.

Assessment scores were analysed for reliability by using two statistical methods commonly used in education and healthcare research - an rWG and ICC (inter-class correlation) [16,17]. Both analyses were performed and compared to try to reduce statistical bias [18]. Firstly, an rWG analysis was run as a measure of inter-rater reliability for each Medi-StuNTS skill element. A score of > 0.7 was deemed significant [8,19]. In addition, an ICC was run in the R studio [20]. This is a two-way mixed average analysis that measures scoring consistency. This was run for each individual simulation as well as each skills element - giving ICC3K and overall, Kappa scores. ICC values less than 0.5 are indicative of poor reliability, values between 0.50 and 0.75 indicate moderate reliability, values between 0.76 and 0.9 indicate good reliability and values greater than 0.90 indicate excellent reliability [21,22].

Usability was assessed by mean observability, i.e., the percentage of observations recorded by assessors [7]. The significance of this was assessed by using a $\chi$^2^ test to establish the extent to which NTS were observed vs. not observed, and the statistical significance thereof. Lastly, acceptability was assessed via an analysis of feedback (Table 1).

**Table 1:** Summary of Data Collected and Type of Analysis Conducted

Evaluation Criterion	Hypothesis	Data Source and Analysis
Construct validity		
-Internal structure: Inter-rater reliability & Inter-class correlation	Near-peer educators trained on the use of the Medi-StuNTS tool to rate NTS, will achieve good inter-rater reliability & inter-class correlation	Ratings data: Within-group inter-rater agreement statistics (rWG) and inter-class correlation (ICC) to show the level of rater scoring consistency/reliability.
-Response process	The Medi-StuNTS system is straightforward for near-peer educators to use to rate NTS	Questionnaire data from assessors
-Consequences	The Medi-StuNTS tool facilitates teaching, learning and changes to clinical practice	Questionnaire data from students and assessors
Usability		
-Observability	The Medi-StuNTS tool is able to identify NTS in medical student simulations, including when assessment is carried out by near-peer educators	Ratings data: Basic descriptive statistics of number of skills observed versus not observed and $\chi$^2^ test to establish the extent to which NTS were observed vs. not observed
-Acceptability	The Medi-StuNTS tool is an acceptable tool for a) assessing and b) training medical student NTS when used by near-peer educators	Questionnaire data from students and assessors: thematic analysis

## Results

Students

Four final-year medical students took part - all had previous experience with simulations that were run in the Oxford simulation suite as part of their medical training. None had ever done NTS-specific simulations before. All students attended our two-hour NTS course.

Quantitative questionnaire data

A summary of student comments can be found in Table 2 - these highlight the tool’s acceptability and provide further evidence of consequences.

**Table 2:** Student evidence of consequences and acceptability of the medi-StuNTS tool

Student Evidence of Consequences and Acceptability of Medi-StuNTS	
Consequences	Key learning points: “Very valuable feedback on both technical and non-technical skills in managing an unwell patient” “Shared mental models at beginning of an emergency scenario in order to formulate a differential diagnosis as a team” Changes to future clinical practice: “Being aware of how sick patients are and escalating earlier” “I will be able to ensure my communication is clear and concise in teamwork environments” “Learning how to speak up to seniors when I am worried about a patient”
Acceptability	Students were grateful for the quality of feedback provided: “very valuable feedback” “useful feedback” “enjoyed the 1 to 1 feedback” “This should be part of the FY1 induction”

Assessors

Assessors were qualified doctors with between three and eight years of clinical experience. All had experience teaching medical students. Only 44.4% (n=9) had previously received training in giving feedback and running a debrief, and none had experience using BMS to assess NTS.

Quantitative data

Table 3 shows high levels of inter-rater agreement across all skill elements for a combined analysis of leaders and followers, rWG>0.8 [7,21,22]. It also shows high levels of skill observability in all skill elements except for coping with stress. Clear differences in skills observed can be seen between the leader and the follower. Overall skill observability increased from 72.40% to 82.14% between the first and last simulation. Across all scenarios, a $\chi$2 test showed that 0.5% of ratings showed no difference between the use of “observed” vs “not observed”, i.e., $\chi$^2^ was not significant.

**Table 3:** Results for inter-rater reliability (rWG) and observability *rwg=1 represents perfect agreement, rwg=0 represents no agreement; **Higher percentages indicate improved level of observability.

Medi-stuNTS- Skill Elements	Inter-rater Agreement ( rWG)*	Mean Observability**	
		Leader	Follower
Gathering information	0.86	96.88%	48.39%
Recon and understand information	0.90	93.75%	61.29%
Planning, preparing and anticipating	0.87	84.38%	58.06%
Prioritising	0.89	93.75%	67.74%
Recognising and dealing with uncertainty	0.91	78.13%	77.42%
Reviewing decisions	0.93	87.50%	70.97%
Establishing a shared mental model	0.90	96.88%	64.52%
Demonstrating active followership	0.91	53.13%	93.55%
Patient involvement	0.89	93.75%	48.39%
Role awareness	0.92	87.50%	77.42%
Coping with stress	0.92	68.75%	54.84%
Speaking up	0.89	68.75%	87.10%
Situation awareness	0.83	94.55%	50.32%
Decision making and prioritising	0.91	85.67%	72.18%
Teamwork and communication	0.88	95.96%	81.80%
Self awareness	0.85	97.08%	58.89%

Table 4 shows the inter-class-correlation analyses when an ICC was run with the first set of matrices designed to give an ICC score per simulation. This was run on R studio, using the irICC package which allows for missing data, i.e., when a rater left a skills element score blank [23]. An overall kappa score was generated with this matrix format - this score was 0.86, indicating good inter-class correlation. P-values were all < 0.05 - where this was taken as statistically significant.

**Table 4:** irICC analysis with R studio showing an ICC3K score per simulation and overall Kappa score L = leader, F = follower. Number of skills elements = 16, number of raters = 9

Simulation	ICC3K	ICC3K Score Interpretation
1L	0.61	Moderate
1F	0	Poor
2L	0.68	Moderate
2F	0	Poor
3L	0.542	Moderate
3F	0.494	Poor
4L	0.422	Poor
4F	0.62	Moderate
Overall	Kappa = 0.86	Good

Table 5 shows the ICC analysis results when the matrices were designed to show an ICC score per skills element. An overall kappa score was generated with this matrix format - this was 0.89, indicating good inter-class correlation. P-values were all < 0.05 - where this was taken as statistically significant.

**Table 5:** irICC analysis with R studio showing an ICC3K score for each skill element Number of simulations = 4 leader & 4 follower, Number of assessors =9.

Medi-stuNTS- Skill Elements	ICC3K	ICC3K Score Interpretation
			Gathering information	0.82	Good
Recognise and understand information	0.98	Excellent
Planning, preparing and anticipating	0.93	Excellent
Prioritising	0.88	Good
Recognising and dealing with uncertainty	0.87	Good
Reviewing decisions	0.88	Good
Establishing a shared mental model	0.94	Excellent
Demonstrating active followership	0.92	Excellent
Patient involvement	0.86	Good
Role awareness	0.95	Excellent
Coping with stress	0.89	Excellent
Speaking up	0.74	Moderate
Situation awareness	0.76	Good
Decision making and prioritising	0.88	Good
Teamwork and communication	0.94	Excellent
Self awareness	0.84	Good
Overall	Kappa =0.89	Good

Qualitative questionnaire data

Assessors initially found the tool difficult to use, however, felt that it provided a useful structure for assessment. They felt that improved familiarity would be useful in their ongoing teaching. Three assessors commented specifically that the reverse scoring system used was counterintuitive. They felt it should be revised so that a higher score indicated better performance. Assessor comments have been summarised in Table 6.

**Table 6:** Assessor evidence of response process, consequences and acceptability of medi-StuNTS (subdivided by theme)

Assessor Evidence	
Response process	Examples of observable behaviours were valued:
	“Useful examples of good and bad practice”
	“Examples of observable behaviours “[helped] pick out key areas”
	“It forces me to note down areas where there are concrete examples of how it went”
	The tool helped identify poor practice:
	“Helps to discriminate poor behaviour”
	“As a tool it is definitely good at discriminating bad behaviour”
	“I was thinking at the end that this is quite useful because when it’s a 5 you know it’s a 5”
	Difficulty using the tool:
	“Tool difficult to use if not very familiar”
	“Not very user friendly”
	“Not very intuitive”
	Assessor overload:
	“a lot of separate scores, hard to complete in time”
	“use audio/visual feedback for review and marking as quite a lot of info to fill [in]”
	“hard to keep track to score both leader and follower”
	Consequences	Improving recognition of NTS:
		“Will have better recognition of NTS when providing feedback”
“I will be better able to recognise non-technical skills within colleagues”	
Providing constructive feedback:	
“Tool allows you to point out good and bad features specifically. This helps make it [feedback] objective and less personal”	
“Useful for feeding back in a constructive way”	
Allows “more structured and comprehensive feedback as assessment is sub categorised”	
Acceptability		75% (n=8) felt it was a useful tool for colleagues to use and that they would implement it in their teaching practice

## Discussion

This study is the first to show that the Medi-StuNTS BMS has good usability and evidence of validity in naive assessors and near-peer educators - it shows particularly good internal structure and overall beneficial consequence.

Evidence of validity

Internal Structure Evidence: Inter-rater Reliability and Inter-class Correlation

Assessment of the Medi-StuNTS tool’s internal structure showed it to have good inter-rater reliability with an rWG >0.8 in all skills elements. This reliability was further tested with an ICC analysis. Overall Kappa scores were > 0.8 but <0.9, indicating “good” inter-rater consensus. It was encouraging that both tests showed “good” reliability and that this was considerably higher than in other BMS validation studies [7,8]. This could be due to the fact that 1) all near-peer assessors were from the same hospital, 2) they were trainees themselves, and 3) many had prior experience in teaching Oxford University final year, medical students.

It was interesting to see that assessors were consistent in being able to identify good and bad performances using this tool, i.e., the ICC was consistently good when run per simulation. When running these simulations again, it would be useful to involve a wider variety of examiner experience and indicate this on the scoring sheets (in this study we had left all mark sheets anonymous). Unblinding this would help us analyse the difference between near-peer vs. more experienced NTS examiners. If it was found that this difference was not significant, it would further support the use of this BMS in the naive assessor setting.

Response Process Evidence

We had initially set out to assess the raters' response process by asking them to note down examples of why they gave specific marks. Analysis of these comments, despite poor completion rate, highlighted difficult tool usability and described the tool as “not very intuitive” (Table 6). In particular, the reverse scoring system seemed to “throw many assessors off” - overall it felt unnatural to give high performing candidates a low score.

Assessors also noted that the focus was on the leader, and that scoring the follower was not done as attentively. The importance of this is discussed further in “Observability of Skill Elements”. In further studies, it would be useful to analyse if improved familiarity with the tool lead to more attentive follower assessment.

Consequences Evidence

We predicted that the beneficial consequences would largely be focused on changes in clinical practice and education going forward, i.e., medical students feeling more confident in using NTS and assessors feeling more confident in assessing NTS. We were surprised to find that in addition to these, there was overwhelming feedback focussed on the debriefing element of the simulation. This tool meant that naive, near-peer educators felt able to provide students with detailed and specific feedback on their performance, which students were appreciative of (Table 2). The assessors felt their feedback was more comprehensive and were more confident and at ease when delivering it - the tool provided structure and objectivity. The students responded very well to the feedback, and their reasons included: 1) comprehensiveness, 2) following an ordered structure, and 3) knowing what to expect. One student nicely summarised the impact this had: “receiving feedback in a structure I was expecting, meant that it felt more constructive and less demoralising compared to previous simulation debriefs”. This “aligning of expectations” is important as it helps establish “psychological safety”, which is essential in order to optimise learning outcomes during debriefing [24]. This is particularly important when using co-workers as near-peer assessors.

Tool usability - observability of skill elements

Over the course of the day, there was a 10% increase in skills observed between subsequent simulation sessions. While this could relate to the simulation scenarios and participants, it is more likely to be a result of increased familiarity with the tool. Assessor training did not provide any practical experience using the tool and it is possible that pre-reading may have not been fully undertaken given busy schedules.

Overall, however, skill observability was high, but did vary between leaders and followers. It was difficult for the leader in each scenario to demonstrate active followership. Hamilton et al, chose to include followership and exclude leadership as they felt that leadership was less relevant given that all medical students would be at the same level of clinical experience [1]. However, research suggests that real life emergencies run more smoothly when an individual leads, even when they are of the same grade [25-27]. Although active followership is a vital skill for junior members of the medical team, it is also important that they are able to lead when required to. This leadership element is often found in BMS, including those targeted at junior grades, e.g., the Foundation Non-Technical Skills BMS tool [28]. We thus feel that the Medi-StuNTS BMS may benefit from integrating leadership into the skill elements.

Hamilton et al. emphasise the point that stress is highly prevalent among newly qualified doctors and including it in the BMS provides a valuable opportunity for discussion and reflection [29]. In our assessment, the skill element “coping with stress” was rarely observed either negatively or positively. This is an inherently difficult skill to assess externally as only the individual themselves can truly assess how stressed they are. While there may be external cues as to how stressed or flustered the candidate is in the simulation, these are often only subtle and may only be noticeable in extremis. Although our scenarios were emergencies and inherently stressful, these did not specifically highlight or exaggerate this element, as we did not feel it was fair to do this to medical students in the given environment. Moreover, we were very mindful that some students shy away from simulations for this reason, and that a very stressful experience can have long lasting, negative effects on how they view simulation training. Going forward, this element could be analysed with planned video scenarios.

Furthermore, video scenario analysis provides the opportunity for score calibration, as demonstrated in other studies [7-9,19]. Video simulations would allow a wider range of scenarios and candidates, which would mean that we could fully assess scoring on some of the less observed skills elements. It would also allow further assessments of inter-rater reliability when scoring poor NTS. It is important to note, however, that while this additional video training may be useful, it would take up time and this is something that medical educators, who are often near-peer volunteers, have in short supply.

Overall, with all the above taken into account, we feel that our study is representative of real-life training, and it is thus reassuring that good utility and reliability can be achieved.

Limitations

This study has a number of limitations. First among these was the size of the study and the fact that participants were from a single institution. Such a small group of self-selected markers may not be reflective of the wider medical education population in this country or in others.

While we are confident that our analyses show good inter-rater reliability and inter-class correlation, the scores should be interpreted with caution for the following reasons: 1) Small number of raters and simulations run, and 2) reduced observability in the followers, leading to many unfilled BMS scores. While the irICC code takes this into account, it may give over-inflated, under-powered ICC scores. Moreover, an rWG is ideally run with at least 10 raters. We chose to run this test, despite only having nine raters, as it was used in the validation of ANTS, which is now well established in simulation training [7]. We applied the Bamford Hill criteria, and compared our data to that in the ANTS study, and overall felt that it would be most appropriate to use the same statistical analysis for our data [30].

As one final point, we used unscripted low-fidelity simulations for logistical and financial reasons. We were concerned that the lack of a more realistic environment would have impacted on the ability of the medical students to demonstrate some key NTS. This was not the case though as a high degree of skill observability was still noted.

## Conclusions

In conclusion, this is the first study to evaluate the evidence of validity and usability of the Medi-StuNTS tool when used by naive, near-peer educators to assess NTS in final-year medical student simulations. The Medi-StuNTS tool has shown good usability, good internal structure, and significant beneficial consequences - both in assessing NTS during the simulation and in delivering constructive debriefs. This opens the door for larger-scale studies to take place in different centres to further validate this tool. This is important as when NTS are examined, these behavioural marking schemes, or “tools of the trade”, need to be robustly assessed and standardised in order to be interpreted meaningfully - especially as NTS becomes more prevalent in medical education and post-graduate medical training.

## Appendices

Appendix A

**Table 7:** Student questionnaires pre and post simulation Students were asked to provide a score between 1 and 10 with a higher score indicating a greater level of agreement with the question. Questions A-G required free text reponses.

Pre-simulation Questionnaire for Students	Answer
1. Have you heard of Human Factors or non-technical skills?	Yes / No
2. How confident are you with your understanding of what Human Factors is?	1-10
3. How confident are you with your understanding of what non-technical skills are?	1-10
4. How important do you think non-technical skills are in everyday life?	1-10
5. How important do you think non-technical skills are in medicine?	1-10
6. How confident do you feel in assessing and managing a very unwell patient	1-10
7. How confident do you feel in your ability to communicate or handover important information efficiently?	1-10
8. How confident do you feel in your communication in emergency situations?	1-10
9. How confident do you feel in leading an emergency situation?	1-10
10. How confident are you in your ability to work well in a team?	1-10
11. How confident do you feel in your ability to recognise your limitations and know when to call for help?	1-10
12. How confident do you feel in your ability to recognise stress in yourself and take action to cope with it?	1-10
Post-simulation Questionnaire for Students	
1. How confident are you with your understanding of what Human Factors is?	1-10
2. How confident are you with your understanding of what non-technical skills are?	1-10
3. How important do you think non-technical skills are in everyday life?	1-10
4. How important do you think non-technical skills are in medicine?	1-10
5. How confident do you feel in assessing and managing a very unwell patient	1-10
6. How confident do you feel in your ability to communicate or handover important information efficiently?	1-10
7. How confident do you feel in your communication in emergency situations?	1-10
8. How confident do you feel in leading an emergency situation?	1-10
9. How confident are you in your ability to work well in a team?	1-10
10. How confident do you feel in your ability to recognise your limitations and know when to call for help?	1-10
11. How confident do you feel in your ability to recognise stress in yourself and take action to cope with it?	1-10
A. What are the most useful things you have taken away from the Non-technical skills Simulations and Debrief sessions?	
B. Did you achieve your learning outcomes?	
C. Please comment on how you will use what you have learnt on the course today in your every day clinical practice.	
D. Did you find the use of simulation helpful in your learning experience?	
E. Would you recommend this training to others?	
F. Is there anything you think could be improved?	
G. Any other comments?	

Appendix B

**Table 8:** Assessor questionnaires pre and post simulation Assessors were asked to completed the above questionnaires which included a mixture of free text responses, Yes/No answers and asking them to provide a score between 1 and 10 with a higher score indicating a greater level of agreement with the question.

Pre-simulation Questionnaire for Assessors	
1. Have you been trained in giving feedback and running debrief sessions for junior trainees?	Yes / No
2. How confident are you with your understanding of what non-technical skills are?	1-10
3. How confident do you feel in recognizing non-technical skills in simulations?	1-10
4. How confident do you feel in assessing non-technical skills in simulations?	1-10
5. What methods do you think would be most useful in training Doctors to assess non-technical skills?	1-10
6. Do you know of any tools or scoring systems used to assess and give feedback on non-technical skills? Have you ever used these before?	1-10
7. How confident do you feel in debriefing and giving structured feedback in general?	1-10
8. How confident do you feel in debriefing and giving structured feedback on non-technical skills in simulations?	1-10
Post-simulation Questionnaire for Assessors	
1. How confident are you with your understanding of what non-technical skills are?	1-10
2. How confident do you feel in recognizing non-technical skills in simulations?	1-10
3. How confident do you feel in assessing non-technical skills in simulations?	1-10
4. How confident do you feel in debriefing and giving structured feedback in general?	1-10
5. How confident do you feel in debriefing and giving structured feedback on non-technical skills in simulations?	1-10
6. How useful did you find the Medi-StuNTS marking tool in helping identify non-technical skills?	1-10
7. How useful did you find the Medi-StuNTS marking tool in scoring non-technical skills?	1-10
9. How do you think the skills you have learnt will influence your day to day clinical practice?	
10. Would you recommend the training to a fellow colleague? Why?	
11. What aspect of the training or the Medi-StuNTS marking tool did you find useful?	
12. What aspect of the training or the Medi-StuNTS marking tool could be improved?	
